# Conducting Social Science Research During Epidemics and Pandemics: Lessons Learnt

**DOI:** 10.1177/10497323231185255

**Published:** 2023-07-04

**Authors:** Jennifer I. Van Nuil, Megan Schmidt-Sane, Alex Bowmer, Hannah Brindle, Mary Chambers, Ragil Dien, Christine Fricke, Yen Nguyen T. Hong, David Kaawa-Mafigiri, Sonia Lewycka, Samita Rijal, Shelley Lees

**Affiliations:** 1 160913 Oxford University Clinical Research Unit (OUCRU), Ho Chi Minh City, Vietnam; 2 Tropical Medicine and Global Health, Nuffield Department of Medicine, Oxford, UK; 3 15592 Institute of Development Studies, Brighton, UK; 4 215824 London School of Hygiene and Tropical Medicine, London, UK; 5 Eijkman Oxford Clinical Research Unit (EOCRU), Jakarta, Indonesia; 6 Translators without Borders/CLEAR Global, Mainz, Germany; 7 308367 Makerere University, Kampala, Uganda; 8 160913 Oxford University Clinical Research Unit (OUCRU), Kathmandu, Nepal

**Keywords:** epidemics, COVID-19, rapid research, Southeast Asia, South Asia, East Africa

## Abstract

The COVID-19 pandemic has had a significant impact on how field-based research is being conducted globally. Given the challenges of undertaking fieldwork during epidemics and the need for mixed methods research to address the social, political, and economic issues related to epidemics, there is a small but growing body of evidence in this area. To contribute to the logistical and ethical considerations for conducting research during a pandemic, we draw on the challenges and lessons learnt from adapting methods for two research studies conducted in 2021 during the COVID-19 pandemic in low- and middle-income country (LMIC) settings: (1) in-person research in Uganda and (2) combined remote and in-person research in South and Southeast Asia. Our case studies focus on data collection and demonstrate the feasibility of conducting mixed methods research, even with many logistical and operational constraints. Social science research is often used to identify the context of specific issues, to provide a needs assessment, or inform longer-term planning; however, these case studies have shown the need to integrate social science research from the start of a health emergency and in a systematic way. Social science research during future health emergencies can also inform public health responses during the emergency. It is also crucial to collect social science data after health emergencies to inform future pandemic preparedness. Finally, researchers need to continue research on other public health issues that are ongoing even during a public health emergency.

## Introduction

The COVID-19 pandemic has highlighted a need for mixed methods research to address social, political, and economic issues related to the pandemic as well as research on public health issues beyond COVID-19. However, the pandemic has had a significant impact on how field-based research is conducted globally. Epidemics and the COVID-19 pandemic constrained the ability of researchers to conduct fieldwork, given the restrictions on travel, social distancing measures, and risk of infection for researchers and participants. These challenges have resulted in postponement of projects, but given the duration of the pandemic, this has not always been practical. Additionally, funders, while flexible to some extent, often required research to be completed within given timeframes. In order to continue existing research, or to initiate research about the pandemic, researchers had to adapt by including innovative methods to collect data remotely (Dodds & Hess, 2021). These included interviews conducted over the telephone, focus group discussions (FGDs), online surveys through the internet or SMS, and self-collected data such as diaries (Hensen et al., 2021).

**Figure 1.**
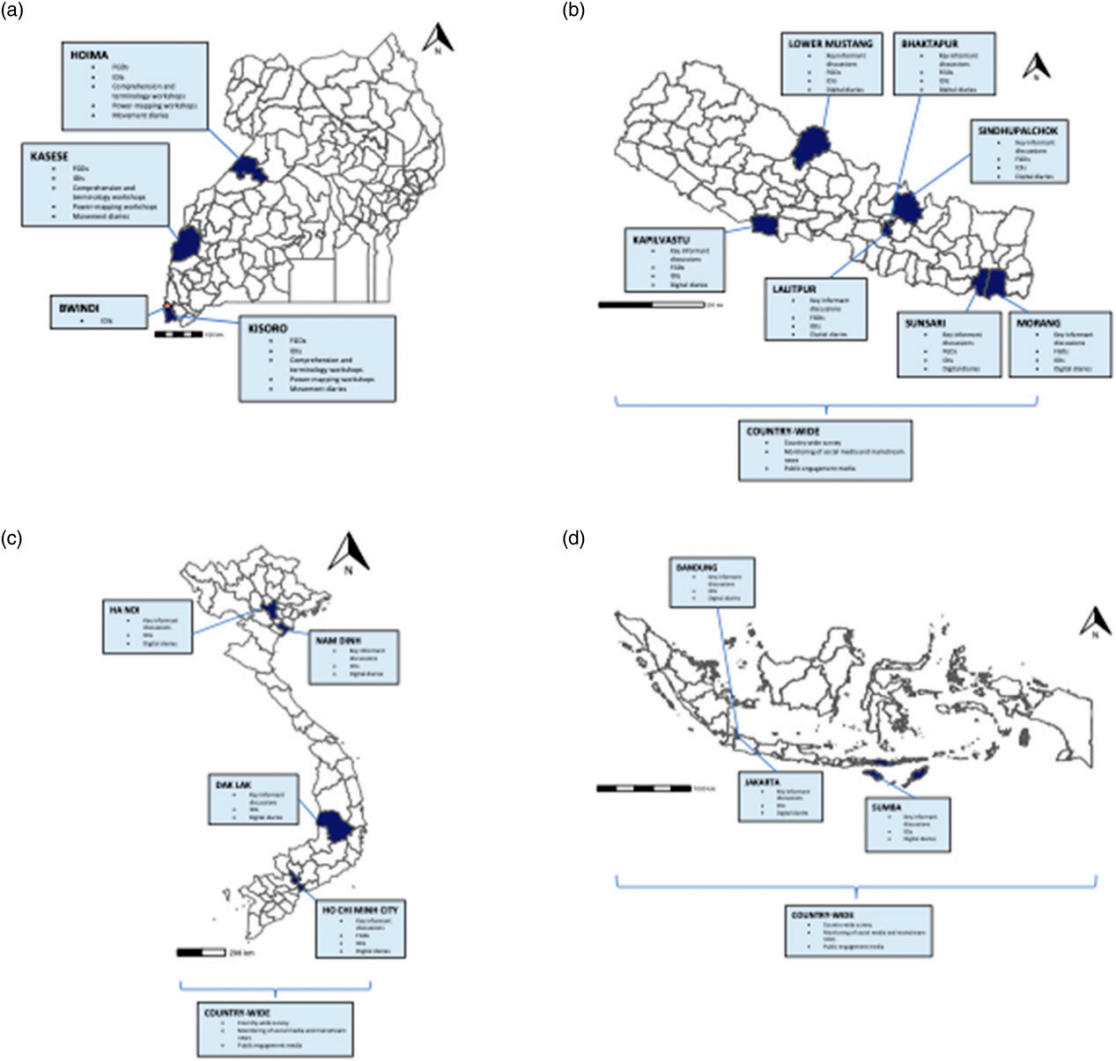
The provinces/districts in which the studies were conducted are shaded in dark blue with the exception of Bwindi, Uganda, the location of which is shown by a pink diamond.

In this paper, we draw on the challenges and lessons learnt from adapting data collection methods for two research studies conducted in 2021 during the COVID-19 pandemic in low- and middle-income country (LMIC) settings (Figure 1): (1) in-person research in Uganda and (2) combined remote and in-person research in South and Southeast Asia. Using these as case studies, we demonstrate how challenges were addressed during the planning and conduct of research.

### Considerations for Using Remote Social Science Data Collection Methods

Given the challenges of undertaking fieldwork during epidemics and the need for rapid and/or remote research to address the social, political, and economic issues related to epidemics, there is a small but growing body of evidence in this area (Johnson & Vindrola-Padros, 2017). Advances in technology including platforms for video conferences such as Zoom and Microsoft Teams (Microsoft Teams, 2021) to some extent enabled this to take place. However, in places where WiFi and phone connectivity is limited, or where large parts of the population cannot access phone or online platforms, these are not viable options. Limited mobile phone ownership amongst poorer populations and limited access to data may contribute to biases in the recruitment of participants by selecting those from more socioeconomically affluent backgrounds.

Hensen et al. (2021) outline issues that need to be considered when conducting remote data collection during epidemics/pandemics. These include sampling, the consent process, participant confidentiality, and the ethical implications of research during a pandemic. Some of these challenges are heightened with qualitative research, particularly in-depth interviews (IDIs) and FGDs which rely on in-person rapport with the interviewer or facilitator. Cornejo et al. (2023) highlight the relational aspects of remote research, especially the emotional impact that research can have on all parties involved. Psychological distress is more difficult to detect during a remote interview, particularly if the participant has their camera off or if non-verbal cues are difficult to see over video. These considerations are discussed in the following sections.

## Case Study 1: Building Trust and Community Ownership of Ebola Awareness and Community Engagement in Uganda (“*Building Trust*” *Study*)

### Background

**Figure 2.**
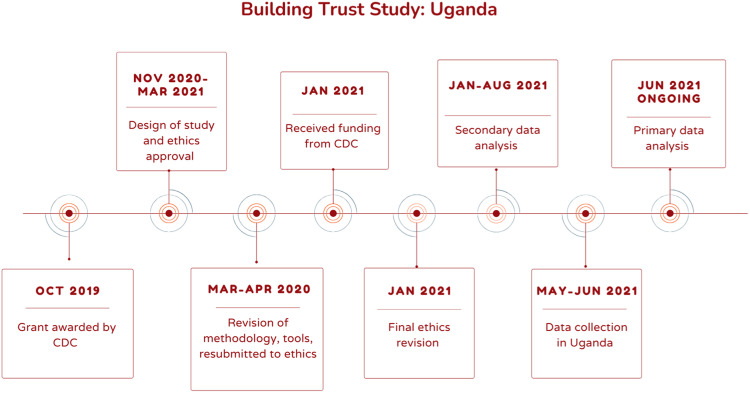
Timeline of Building Trust Study: Uganda.

The “Building trust and community ownership of Ebola awareness and community engagement in Democratic Republic of Congo (DRC) and Uganda” study (“Building Trust” study) aimed to address challenges in establishing trust for effective care and containment of Ebola virus disease (EVD) in a context of cross-border and within-country mobility and associated risks in the wake of the 2018–2020 EVD epidemic in Democratic Republic of Congo. Uganda saw four cases of EVD during this time, primarily linked to cross-border movement from the DRC. The study included secondary data analysis of work in DRC and primary data collection in Uganda, and so we only present methods used in Uganda in this paper. Our study pivoted to also address COVID-19 preparedness and response. The study used a rapid mixed methods research design using predominantly qualitative methods. It also aimed to develop operational guidance for implementing partners to inform current and future epidemic response and preparedness activities in addressing (mis)trust in practice. This was a collaborative project between the London School of Hygiene and Tropical Medicine, UNICEF, Makerere University, and CLEAR Global/Translators without Borders (Figure 2). The data collection period commenced during the peak of the COVID-19 pandemic (April–June 2021).

### Original Methods

In this project, we initially planned to use mixed methods to explore five themes which would provide information about the challenges in establishing trust for the effective care and containment of Ebola only in the context of cross-border and within-country mobility. In light of the pandemic, we included questions around COVID-19 for these five themes. Overall, study themes included Communication; Health-seeking behavior; Trust in authorities; Population movements; and Hunter/scavenger behaviors. We conducted the following during datacollection: 15 in-person FGDs (79 participants), 71 IDIs, 3 power mapping workshops, community walks and observations in 3 districts in western Uganda and 25 interviews in Bwindi. We had intended to ask a subset of participants to complete a movement diary whilst simultaneously wearing a global position system (GPS) device, but due to the public health measures, we only conducted 36 movement diaries without the GPS device. Participants were purposively selected based on border groups considered “vulnerable” to EVD and/or COVID-19 or to the effects of public health measures. These included long-distance transport workers, motorcycle taxi drivers, fisherfolk, border market and informal traders, female sex workers, Batwa (indigenous people), and reformed hunters.

### Adapting the Field-Based Research Methods in Uganda

This research went through several planning iterations in light of rapidly changing local and international travel guidelines. During the early phases of the COVID-19 pandemic in spring 2020, it became apparent that conducting in-person research would be logistically challenging due to both in-country and international travel restrictions, in addition to ensuring the safety of participants and staff. At this point, we made plans to conduct the research remotely. However, a decline in COVID-19 cases in summer 2020 allowed for the relaxation of some restrictions, including local travel within Uganda. As teams were able to move to the field sites, it was possible to gather in small groups of less than 20 people while maintaining social distance and utilizing masks and sanitizer/hand washing facilities and in-person IDIs and FGDs were now possible. As international travel restrictions relaxed, in early 2021, our international team members were able to travel to Uganda and participate in the local onsite activities together with team members in-country. However, we had to adapt the methods to COVID-19 protocols and restrictions, which included some restrictions on cross-border movement. Therefore, the use of GPS devices was not feasible.

### Use of Technology in Data Collection and Management

Data were collected in Uganda using the open-source software Open Data Kit (ODK) (ODK—Collect Data Anywhere, 2021). Open Data Kit allows off-grid data collection with end-to-end encryption. This enabled us to work in areas without internet access whilst complying with General Data Protection Regulations (GDPR). XLSForms were developed to allow (1) FGDs and IDIs to be recorded using an in-built recording system and (2) movement diaries to be completed using a survey format, both within the ODK Collect application downloaded onto Android devices. Within the movement diary, GPS coordinates of the locations visited could be obtained through the use of an inactive map.

Once internet access was available, all forms were synchronized to a central server based at the London School of Hygiene and Tropical Medicine. Data was then downloaded and decrypted from the server from any location where internet access was available, providing a real-time secure data-sharing platform. The use of ODK was helpful in terms of collecting data, particularly demographic forms and movement diaries. Despite training, recording in ODK proved challenging for some field staff, who reported data loss. We had a few cases of phone recordings malfunctioning, and some research assistants (RAs) had more trouble using ODK than others. Internet connectivity in the field was variable, and we often relied on uploading/downloading files when we returned to our district home base.

At the time of the project, the ODK central server acted as a unidirectional system, permitting the download of data only. Therefore, de-identified datasets, transcripts, and analysis codes were uploaded to Microsoft SharePoint where these could be securely shared between team members.

### Logistics and Ethical Challenges

We obtained ethics approval from Makerere University, Uganda National Council for Science and Technology, and the London School of Hygiene and Tropical Medicine. However, given the changes in study design due the pandemic, we requested amendments to the protocol. Fortunately, these were reviewed rapidly by both committees, which did not result in any delay once the protocol was finalized. In hindsight, flexible protocols would have allowed for the possibility of conducting either in-person or remote data collection in unpredictable situations. Our team had both quantitative and qualitative skills, but with a mainly qualitative protocol, we had more flexibility built in, to account for changing realities “in the field.” Expanding this flexibility would have made the pandemic challenges easier to manage.

Obtaining consent remotely posed a number of challenges when we considered planning for remote research. These included how to verify that verbal consent was obtained without a signed consent form, and how to determine whether participants fully understood the information provided during the consent process. Ultimately, we were able to obtain consent in person, where the researchers were able to gain more insight into the comprehension of the participant, for example, by reading facial expressions.

### Analysis Challenges

Despite using ODK, which has in-built validation for data collection, there was still a large amount of data cleaning to be done for the movement diary data. This was in part due to the requirement to record participant details in a separate form which resulted in some discrepancies between participant IDs. The use of electronic data collection for longitudinal data would have helped to mitigate this issue. Additionally, whilst we used a “cascading selection” system for documenting geographical locations, we were still required to clean an abundance of free text. This was the result of not using up-to-date information relating changes to the names and divisions of administrative zones. In the future, we would consult further with local staff prior to data collection to avoid this problem and engage in more robust piloting of data collection.

## Case Study 2: COVID-19 Social Science and Public Engagement Action Research Study

### Background

**Figure 3.**
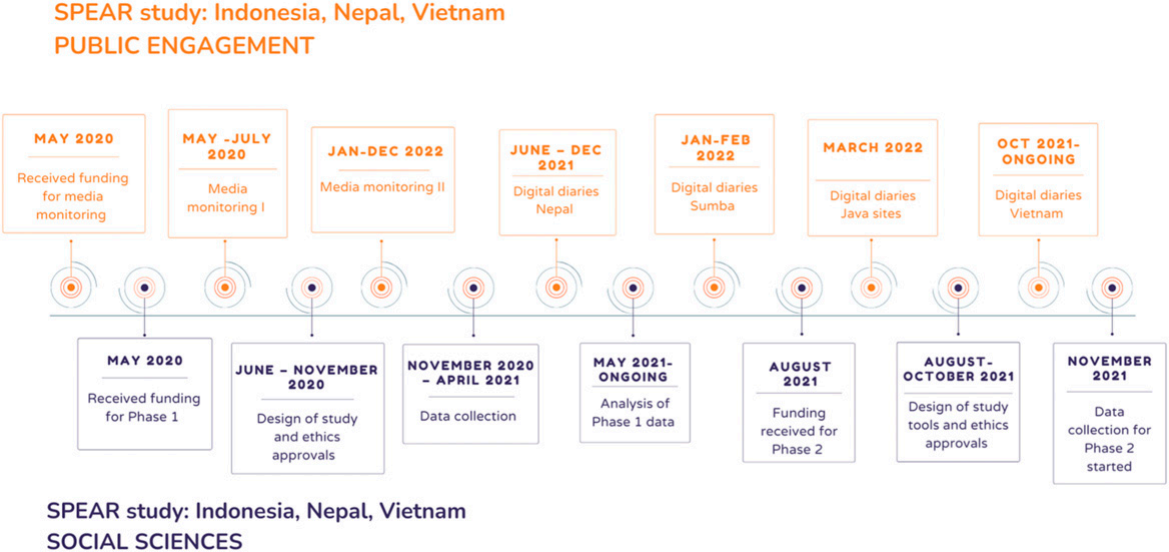
Timeline of SPEAR study, all components. Note: orange boxes represent Public Engagement activities and dark blue represents social science research.

The COVID-19 Social Science and Public Engagement Action Research (SPEAR) in Vietnam, Indonesia, and Nepal is a multi-country study using multiple methods to explore the experiences and impacts of COVID-19 for healthcare workers (HCWs) and vulnerable communities, including participants from 13 Oxford University Clinical Research Unit (OUCRU)–related sites across Indonesia, Nepal, and Vietnam (Van Nuil et al., 2021). Data collection took place over a period of 24 months from September 2020 until August 2022, and analysis for some components is ongoing (Figure 3).

### Original Methods

There were two main phases of inquiry that followed the course of the pandemic at the study sites. In the first phase, we were concerned with lived experiences, work life and livelihoods, new or deepened vulnerabilities, and aspects of mental health and coping for the participant groups. In the second phase, we focused on vaccination, including acceptance of and access to COVID-19 vaccines, as well as factors that may influence vaccine acceptance and access. We combined survey methods and social media monitoring with IDIs and digital diaries to provide both general and specific pictures of the situation within each study site. Study sites included a range of clinical settings and both rural and urban communities to gain a wide range of experiences. Participants included (1) HCWs and related healthcare staff based on position (nurses, doctors, cleaners, and drivers), healthcare facility level (local health centers to national hospitals), and years of experience and (2) community members from groups who may be more vulnerable due to COVID-19 and/or the public health response (e.g., elderly populations, recovered COVID-19 patients, and migrant workers).

The original methods included a minimum of 400 surveys per country for both HCW and community groups, 10–15 IDIs per site, 1–2 key informant discussions (KIDs) per site with multiple discussions throughout the project, 1–2 FGD at one hospital site, and up to 15 digital diaries per country.

### Adapted Methods for Fieldwork in SPEAR Project Sites

We designed the SPEAR project during the early stages of the COVID-19 pandemic when the realities were quite different in each research site. The main challenge in planning the study was that the situation was rapidly evolving, so flexibility was extremely important for this study. Due to widespread uncertainty and the diversity of public health responses, we wrote a flexible protocol to allow for site- or country-specific data collection processes and study topics including flexibility in the number of interviews per participant, the format of interviews (e.g., online or in person), and the format of survey (e.g., online or paper self-administered and on phone or in-person interviewer-administered). The participant-led digital diary format was should be varied by site depending on popular local media platforms and available technology. We added KIDs as a method in case we were unable to have a researcher in all the sites, at all times. The key informants helped to identify participants for IDIs, help us think through the meaning of “vulnerability” within specific sites and if/how that changed over time, and to identify key issues related to COVID-19 within their communities. In Indonesia, we conducted the KIDs as we disseminated the research plan with our local partners including government, hospital, health offices, and community leadership, which helped to build a sense of local ownership. In Nepal and Vietnam, we conducted the KIDs at the start of the study with leadership from national hospitals (e.g., Patan Hospital in Kathmandu and Hospital for Tropical Diseases in Ho Chi Minh City), as well as community leadership in Nepal, prior to data collection as well as over the course of the study.

In SPEAR Phase 1, we conducted a total of 221 IDIs. In Indonesia, of the 70 interviews, 55 were conducted online and 15 in person. In Vietnam, we conducted a total of 79 interviews, with 9 online and the remaining 70 in person. We also conducted two FGDs at one hospital site in Vietnam. In Nepal, we conducted 16 interviews online and 58 in person.

In Vietnam, the researchers conducted only one interview per participant, but in Indonesia and Nepal, several participants had more than one interview session due to the participants’ availability and/or to avoid long phone discussions. We also found that some participants preferred online interviews. Overall, having flexibility allowed us to adapt to the situation in each site.

### Use of Technology in Data Collection and Management

We kept flexibility in data capture methods used because of pandemic-related movement restrictions, as well as differences in the characteristics of the participants we wanted to reach. We used a REDCap server (Harris et al., 2009, 2019) and electronic data capture platform, which allowed us to disseminate the survey as an online self-completing questionnaire, or for interviewers to conduct interviews over the phone or face-to-face and capture data directly into REDCap, or for interviewers to capture responses on paper and enter data into REDCap later. Face-to-face interviews were particularly important for specific vulnerable groups who could not complete surveys on their own due to low literacy, poverty, or remoteness. However, this meant that potentially the data collectors might influence the responses while assisting the participants or to data not being collected at all as the workload for those on the ground was too intense. Using REDCap also raised technical challenges, as this platform was new to the data team. Technical problems were dealt with as they arose. It was particularly challenging to develop and test the survey in four different languages and ensure that the meaning was consistent. We reviewed and piloted each tool several times before finalizing it.

When it was not possible to conduct the IDIs in person, we tried to use a platform that would allow for video calling so that the interviewer could see the participants and feel more comfortable. If this was not possible, we collected the interview data via phone with no video. The recommended platform for remote interviewing, according to our institution, was either Zoom or Microsoft Teams, but we rapidly learned that most of the participants (if not all) did not have access to these technologies.

### Logistics and Ethical Challenges

The restrictions on travel posed challenges to setting up the research teams in three countries. We primarily used remote contact for job interviews, for orientation sessions, and for training. We integrated routine calls, often multiple per week, to keep teams connected at both country and full-team levels, although this led to the teams spending many hours per week on study calls. Throughout the study period, teams would move in and out of working from home as restrictions changed. Some study staff were hired and started working without visiting the office or team in person for weeks or months. While this was challenging for specific sites and individuals, it was an advantage for the full SPEAR team as our norm was to meet online; therefore, the meetings were much more inclusive and staff in remote locations were not disadvantaged.

We conducted the survey and REDCap training both online and in person for data collectors who were outsourced from the local sites. We conducted all the qualitative training and debrief sessions after interviews online with the full SPEAR team.

The study was reviewed and approved by the National Hospital for Tropical Diseases Ethics Committee (Hanoi, Vietnam), Hospital for Tropical Diseases Ethics Committee (Ho Chi Minh City, Vietnam), Ethics Committee of Nepal Health Research Council (Kathmandu, Nepal), Patan Hospital Ethics Committee (Kathmandu, Nepal), Ethics Committee of the Faculty of Medicine, University of Indonesia (Jakarta, Indonesia), and Oxford Tropical Research Ethics Committee (Oxford, UK). We also obtained local government permission as required in each context. The logistical challenges of gaining ethics approvals varied across the sites but included issues surrounding payments, distribution of documents, and ethics committee meetings being postponed or canceled, and some committees were not immediately ready for reviewing research protocols remotely. Additionally, in Indonesia, the local governments were more cautious about providing ethics approvals as there were many applications for COVID-19-related research. These factors added delay to the set-up process across sites. However, the amendment process to add Phase 2 components was straightforward in Indonesia and Vietnam as we already had an ongoing study, partnerships, and approvals in place. In Nepal, the ethics committee did not approve the submission as an amendment; therefore, we had to submit it as a new study.

The delays in approvals, among other issues, led to different start dates across sites as we also had to balance our partners’ expectations. For example, in Vietnam, we conducted the HCW component with our partners as soon as we had the national approvals in place. We started to collect qualitative data at this site and added the survey component when the other sites had approvals in place.

### Analysis Challenges

We were working in four main languages, as well as several local dialects; therefore, during survey development, we encountered challenges regarding language and meaning. We back-translated English versions into each language and had multiple sessions to check consistency of meaning across translations.

We started to collect the digital diaries in Nepal before the rest of the study data collection began. We used the scripts from the digital diaries to inform our initial data collection tools. We also held weekly debrief sessions with the full study team. We used these sessions to rapidly disseminate information we were learning from the interviews and social media monitoring, to feed into ongoing engagement activities, as well as to add additional topics across the study sites. We also fed the information from the debrief sessions into the unit-wide OUCRU COVID-19 meetings.

We faced the challenges of conducting qualitative data analysis across languages and contexts. For example, do we all code in one common language or do we code in the languages in which the interviews took place? In the end, we transcribed all the interviews into language spoken and translated the files into English. This added considerable time to the analysis process.

## Reflections Across Sites and Case Study Comparison

### Participant Selection and Recruitment, Including Consent

While the social science research depended on purposive or snowball sampling, we learned early on that these sampling techniques would need to be adapted to the challenges of the COVID-19 pandemic. For the Building Trust Study, we designed multiple strategies for both in-person and remote data collection to reflect the changing situation, although this took a lot of time and effort from the study team. For example, Uganda went into “lockdown” beginning April 1, 2020, that restricted all movements within the country, including the use of private vehicles and public transportation. In our research design, we focused on vulnerable populations in the border context, including long-distance transport workers and market traders among others. It would have been difficult to conduct sampling without on-the-ground support.

To conduct in-person data collection in May 2021, we adapted our protocols to include COVID-19 safety and prevention measures. These had to be approved by local and international ethics review boards. At the time of data collection in Uganda, COVID-19 cases were very low both in the capital (Kampala) and the border districts. We held an in-person training in late April with RAs at Makerere University’s School of Social Sciences with COVID-19 prevention protocols.

For the Building Trust Study, once we arrived in-district, we used a multi-tiered approach to purposive sampling. We collected data in three border districts: Hoima, Kasese, and Kisoro. We consulted with District Health Officers and teams to understand vulnerable sites in the context of previous epidemics. We selected sites where our target populations lived, including landing sites at lake borders, border crossing towns, and transport stops. We used a dynamic approach to this sampling, visiting a different site each day, but also allowing flexibility in case another site was identified as important during our fieldwork. Once we reached a specific site, for example, a landing site, we first met with local leaders and Village Health Team workers who provided critical support to our research. They would assist us with identifying participants based on our criteria (occupation, age, and gender). We paired this with community walks and other sampling based on our own networks in the site where we could identify potential participants in a “more random” fashion. We found that community walks provided context to our study and enabled us to speak informally with a wider population of the site.

For the data collection that took place in Bwindi, due to the sensitive nature of bushmeat collection and movement, the project lead worked with a local non-governmental organization (NGO) to assist with participant recruitment. Community mobilizers and NGO staff selected a small subset of reformed hunters from Bwindi Impenetrable Forest using snowball sampling. Owing to the practice of bushmeat poaching and scavenging being illegal in Uganda, there are only a small number of groups of reformed hunters in this area that are willing to talk about their experiences after being released from prison. The justification for sample size rested on the availability of participants and adequate number of IDIs to gather sufficient information to map bushmeat movement and health awareness between the DRC–Uganda border. Ongoing qualitative research in this area under another related project suggests this sample size is adequate for the purposes of this study.

For the SPEAR KIDs, we selected participants who were already part of our network or a direct referral from someone within our network. Key informants typically included participants in leadership positions at the study sites. For HCW interviews, we wanted to include participants from a wide range of professionals within the study sites to gain a wide range of experiences; therefore, we used a purposive sampling framework and included participants ranging from nurses and doctors to those in managerial roles, to those with non-patient care roles, such as cleaners and drivers and including a variety of ages, experience, and gender. At the sites in Indonesia, we selected participants for IDIs from survey respondents, based on the above criteria. At other sites, we recruited participants through the key informants or other contacts established within our networks.

We planned to use a combination of purposive and random sampling for the SPEAR survey. We wanted to target vulnerable population groups who might be missed in some of the major global online surveys, due to lack of access to smart devices or unstable internet. We also wanted to collect population-level data through random sampling, in order to compare vulnerable groups with the general population. For this, we planned to disseminate links for online surveys through social media as well as sample from the general population in some sites. For both sampling approaches, we relied on our local partners and existing study networks to identify participants and encourage them to respond to the SPEAR survey. This was difficult to implement in some sites because of movement restrictions. In practice, our social media promotion of the survey was not as successful, and we only reached small numbers of the general public in this way. Most survey participants were those targeted purposively from specific vulnerable population groups.

### Embeddedness of Teams in Community

We implemented the SPEAR study at multiple sites in Indonesia, Nepal, and Vietnam, because the OUCRU network was already an established institution in these settings, and we had ongoing research with a range of health facilities and communities. This meant that our research team already had infrastructure and many relationships and partners in place from previous research projects. The development and implementation of SPEAR was feasible because of these preexisting, solid relationships, long-standing collaborations, and knowledge about the local contexts. The broader understanding of the local contexts and cultures also helped us to collect more site-specific data. While initially challenging, having the team embedded across the three countries has led to more specific research questions and a variety of experiences and impacts related to COVID-19. However, our local partners also needed to build trust with our team and the research institute. In a few cases, the trust was lost due to partners not delivering what they promised or rapidly changing our research plans to accommodate their requests. While similar situations have minimally occurred in the past with some partners, during COVID-19, both non-delivery and changing plans also became part of data collection in early phases of the pandemic.

We had similar experiences with research in Uganda, where both collaborations had existed for a long time. Indeed, the project’s RAs had conducted work in the border region on a variety of projects over a number of years. Their fluency in the local languages and knowledge of the cultural and political–economic context proved invaluable, yet they were also somewhat considered outsiders as many were coming from the capital city, Kampala. From the two international staff who conducted fieldwork with this team, one (MS) had conducted work in Uganda for several years and relied on her knowledge of local context. Both (MS and CF) had anthropological training. Further, we conducted a 3-day training session prior to fieldwork, during which time RAs discussed and compared experiences of the local contexts. During our time in the field, we met twice a day and continually reflected on our positionalities, how that might affect the research and the findings, and discussed what we were learning. Our ability to adapt regularly was crucial.

### Building Rapport With Participants Through Remote or In-Person Data Collection

While there were practical and safety reasons for flexibility with data collection formats, there were challenges in building trust with communities and potential participants for the data collection formats that did not include a face-to-face component. For example, at an urban site in Indonesia, there was a site where it took weeks to initiate a partnership. The pre-COVID process would have entailed an in-person visit with the partners and the research team. The partnership was typically then acknowledged by the leaders and approved within days. During the SPEAR start-up phase, in-person visits were not allowed and therefore the communication had to be done remotely. The process to gain the rapport and approvals to conduct research was much longer than before, leading to delays in data collection at that site. Beyond study start-up, we faced similar issues during data collection. For example, in Nepal, based on prior research experiences in these communities, we acknowledged that data collectors from outside the community were typically not accepted, and therefore, remote interviews from people outside the community were also not entertained.

In some instances, in the SPEAR study, we had to balance building trust with data quality. For example, in Nepal, because travel was not allowed when data collection first started and our team was physically based in Kathmandu, we decided to outsource the data collection for the rural and mountainous areas for Phase 1. The SPEAR Nepal team thought it would be best for data collectors from the communities to conduct the IDIs and community surveys face-to-face instead of conducting interviews remotely because the team felt that the trust with participants from in-person data collection would be essential. However, the full SPEAR team was not able to engage in the data collection training and data collection process (e.g., debrief sessions with the full team), at these sites to the extent that we did with the other sites. The IDIs conducted were less detailed and focused resulting in the team in Kathmandu calling the participants to probe for more information to enhance the quality of the interviews. The surveys collected, however, were of good quality based on a review of the responses. For Phase 2, we adapted the process so the team in Kathmandu conducted the IDIs for all the sites remotely, but we used community contacts to make initial connections and refer participants for interviews. The surveys were conducted using the data collectors from the community as in Phase 1.

Other challenges with collecting the IDIs included (1) building trust and connecting with participants was difficult as many participants joined the interview via phone or did not want to turn on camera due to internet connection or shyness or according to interviewers in Indonesia, perhaps participants did not want to show their living conditions; (2) it was not always possible for participants to find a private space for online IDIs; (3) some participants did not pay full attention to the interviews or they were more likely to end the interview compared to the face-to-face format, so at times, the quality of the online interviews was substandard; and (4) finally, some participants did not want to discuss some of the topics, for example, access to PPE, as it was too sensitive within the context. For example, one interviewer who faced this challenge stated that these participants, often at managerial positions within the healthcare setting, likely did not want to report that PPE was not widely available as it might demonstrate a weakness of the healthcare system.

For the Building Trust Study, because we were focused on vulnerable groups for the Uganda data collection, building trust in face-to-face recruitment and data collection was critical. Research assistants played a vital role in this. During recruitment of the research team, we made sure to recruit RAs that the Uganda leads (DKM and MS) had worked with before, so their ability to build trust in the field was known. The RAs were truly key to establishing participant trust in the face-to-face data collection.

### Infection, Prevention, and Control Measures—Risk Mitigation and Mental Health Impact

We adapted the methods to be flexible to make sure that the study teams were conducting the research with minimal risk of contracting COVID-19, among other practical reasons. This involved the option for online interviews, but this was not always feasible for some participant groups who did not have access to such technology. We had to balance data collection format with the risk of COVID-19 transmission, which was not always a clear decision. In some contexts, it was possible, according to the regulations, to conduct interviews in person, but it was not always safe for the researcher and for the research participants. We conducted interviews outside when possible, but also needed to balance protecting privacy with infection prevention best practices.

Delivering a new research project in an extremely uncertain context also added extra stress for the study teams. We faced challenges related to how to best provide mental health support for participants not only because of the study topics but also because of the general COVID-19 situation. At the start of the study, we questioned at what level should and could we intervene and offer support for participants. Before the study started, each team developed a referral process for participants and staff to access mental health support. For example, at one site in Vietnam, this was providing a separate phone number for HCW participants to call and this number was offered to all HCW participants across the country.

The pandemic and restrictions changed throughout the study period and across the 13 SPEAR sites, so as a team, we faced issues regarding health, illness, and tragedy as we progressed through the study. The Building Trust Study team faced the same challenges. There were times when researchers were not able to join the meetings, due to mandatory testing, COVID-19-related site meetings, or other emergencies. We also had team members who had family members pass away during the study, and others whose family and close friends became seriously sick. These events affected team members’ ability to work, and their mental health. In addition to conducting the study, we also had to find sources of support for team members.

For the Building Trust Study, we developed a very detailed COVID-19 protocol that we applied throughout the research. For example, we gave particular attention to the spaces used for research activities, making sure they were outside or in spacious and well aired rooms. For all activities, including training and team briefings, we provided hand sanitizer, masks, and gloves. We also reflected on how to do this in a sensitive way, without creating further barriers between the researchers and participants. For example, the team agreed that the so-called “temperature gun” should only be used to measure temperature at the wrist and should not be held up against a person’s forehead. While we initially worried about how to handle situations in which participants would be reluctant to follow protection measures, participants expressed their understanding and also gratitude that we followed these measures so strictly, including the research teams. In fact, it was us, the research team from the capital Kampala, and two international researchers that were perceived as the epidemiological threat to the community since COVID-19 is considered and was backed by official infection rates as a disease of non-Africans and the urban population in Kampala.

Our COVID-19 protocol impacted the activities in several ways. Setting up focus groups took longer as we needed to explain the risk mitigation measures to everyone. We needed detailed planning of how to implement the protocol, including calculations of the number of masks or gloves needed, and we would often find ourselves running to the local pharmacy to purchase more. Sanitizer, masks, gloves, and thermometers are not easily available in more rural areas and needed to be purchased in larger quantities in the capital or at least regional towns. On top of this, the national COVID-19 protocol further impacted research logistics, including a curfew and travel restrictions and regulations.

## Discussion

This paper presented the methods adapted to the COVID-19 pandemic for two case studies which adapted protocols to allow both in-person and remote data collection in the context of widespread restrictions on movement (see Table 1).

**Table 1.** Comparing Original and Planned Methods to the Modified Ones and Lessons Learnt.

Main Topic	Adapted Methods	Consequences	Lessons Learnt
Format of data collection and the importance of flexibility	Online versus remote data collection	Ethics amendments required	Integration of flexibility into protocols to reduce the amendment procedure
		Different consent process required for remote data collection	Integration of remote consent taking process into SOPs
		Extra training on remote data collection for in-depth interviews	Remote interviewing part of standard training at study start
		Some participants could not be reached using remote data collection and therefore in person required	Central role of local collaborators and having embedded teams at research sites, who co-produce the research throughout all phases
		Challenges of building rapport prior to remote data collection	
Limited travel for study teams and investigators, for various periods of time	Online recruitment and trainings for teams	More integrated teams across sites as all trainings were conducted collaboratively	Full study team trainings and debrief sessions are useful for the team as a whole
	Local data collectors trained in person in local languages	Slightly different understanding of study protocol and methods	Ensure consistency in training material
Recruitment and sampling of “vulnerable” populations	Integration of multiple recruitment and sampling strategies	Easier to adapt across contexts and current pandemic situations	Flexibility enhances recruitment of populations during emergencies but also misses some populations

While social science research has been used to identify the context of specific issues that need to be addressed, provide a needs assessment, or inform longer-term planning (Johnson & Vindrola-Padros, 2017), these case studies have shown the need to integrate social science research from the start of a health emergency and in a systematic way. This includes planning data collection in response to the realities on the ground (Johnson & Vindrola-Padros, 2017; Lees et al., 2020; Luciani et al., 2021) as well as informing public health responses during the course of, and after, the emergency to guide future pandemic preparedness.

Our case studies demonstrate the feasibility of applying methodologies for rapid qualitative data collection despite logistical and operational constraints which suggest that social science research may be *more* suited to uncertainty. The main challenges encountered were related to ethics including prolonged submission processes and obtaining consent remotely, and the changes in the use of technological systems for data collection and recruitment. However, we noted the importance of flexibility in many aspects of study design, implementation, and analysis and would therefore advocate for more flexible protocols and ethics review during future emergencies, which are often uncertain in nature. Delays in starting the Building Trust Study might have been mitigated by allowing oversight of the research by the Ugandan Institute, who had better insight into the current situation and were not impacted by international travel restrictions, rather than the Global North who were largely working remotely.

While it may be difficult to move all qualitative research components online, we offer a few suggestions to adapt the process and methods, using some remote forms with some in-person forms. Similarly, Lobe and colleagues (2020) reflected on “socially distant” methods and weighed the benefits of remote data collection strategies (Lobe et al., 2020). They also flagged additional ethical concerns, as privacy issues are inherent to online data collection (Lobe et al., 2020; Newman et al., 2021). Another consideration is that weighing the benefits and risks of conducting remote data collection compared to in-person research becomes more apparent as restrictions are eased (5). Therefore, it is not always apparent which methods will be more or less successful.

While flexibility tends to be an important aspect of qualitative research in general, flexibility coupled with extreme pandemic uncertainty was challenging. The SPEAR project wanted to initiate data collection as rapidly as possible to ensure the research topics were still relevant; however, this was not possible, due to the many challenges outlined. In the Building Trust Study, we faced similar challenges and modified our data collection tools a number of times to reflect these changing realities. Additionally, using a semi-structured (rather than structured) guide allowed us to build in additional flexibility.

The adaptations and flexibility led to a few losses including missing out on enrolling “new” population groups. The nature of the pandemic and hence restrictions limited the ability to enroll participants who may have been particularly vulnerable for the SPEAR project given that we limited to working within our network where we had a pre-established relationship and hence, trust. However, this trust from our embedded teams enhanced recruitment of populations among those preexisting networks. There were also challenges with staffing. In Uganda, some of the social science teams that had been trained prior to the pandemic and therefore had established knowledge of the research sites had to find alternative sources of work, whereas others were unable to move during the restrictions.

Finally, we need platforms to optimize the collection and sharing of these data. The platforms should also be simple for both data collectors and participants to use while maintaining a high level of data security. Future studies should include development and team training of these technologies to make data collection, sharing, and analysis more convenient but also secure.

## Conclusion

This paper has reviewed methodological challenges and adaptations from two studies conducted during the COVID-19 pandemic across diverse settings. We included a discussion of challenges to participant selection and recruitment, data collection, and staffing during the pandemic. Building on the work of other social scientists who use rapid research methodologies, we reflect on adaptations in methodological approaches with the aim of showing the feasibility of rapid and adaptable social science research despite those challenges. In doing so, we contribute to the evidence base on rapid qualitative and social science research during public health emergencies with the aim of arguing for more systematic integration in public health efforts in the future.
